# Development and formative evaluation of patient research partner involvement in a multi-disciplinary European translational research project

**DOI:** 10.1186/s40900-020-0178-7

**Published:** 2020-02-19

**Authors:** Rebecca Birch, Gwenda Simons, Heidi Wähämaa, Catherine M. McGrath, Eva C. Johansson, Diana Skingle, Kerin Bayliss, Bella Starling, Danielle M. Gerlag, Christopher D. Buckley, Rebecca J. Stack, Karim Raza, Marie Falahee

**Affiliations:** 1 Institute of Inflammation and Ageing, College of Medical and Dental Sciences, University of Birmingham, Birmingham, B15 2TT UK; 2 Rheumatology Unit, Department of Medicine, Karolinska University Hospital and Karolinska Institutet, SE-171 76 Stockholm, Sweden; 3 Department of Rheumatology, Sandwell and West Birmingham Hospitals NHS Trust, B18 7QH, Birmingham, UK; 4 EuroTEAM Patient Research Partners, Stockholm, Sweden; 5 EuroTEAM Patient Research Partners, London, UK; 6 Public Programmes Team, Central Manchester University Hospitals NHS Foundation Trust, University of Manchester, Manchester Academic Health Science Centre, Manchester, UK; 7 Department of Clinical Immunology and Rheumatology, Amsterdam Rheumatology and Immunology Centre, Academic Medical Centre/University of Amsterdam, Amsterdam, Netherlands; 8 RxCelerate Ltd, Babraham Research Campus, Cambridge, CB22 3AT UK; 9 Kennedy Institute of Rheumatology, University of Oxford, Headington, Oxford, OX3 7FY UK; 10 National Institute for Health Research (NIHR) Birmingham Biomedical Research Centre, Birmingham, UK; 11 Division of Psychology, School of Social Sciences, Nottingham Trent University, Nottingham, NG1 4BU UK

**Keywords:** Patient and public involvement, Evaluation, Translational research

## Abstract

**Plain English summary:**

Patient and public involvement (PPI) improves the quality of health research and ensures that research is relevant to patients’ needs. Though PPI is increasingly evident in clinical and health services research, there are few examples in the research literature of effective PPI in translational and laboratory-based research. In this paper, we describe the development and evaluation of PPI in a multi-centre European project (EuroTEAM – **T**owards **E**arly biomarkers in **A**rthritis **M**anagement) that included both translational and laboratory-based and psychosocial research. We found that although most PPI in EuroTEAM was centred around the psychosocial research, there were examples of PPI in the laboratory studies. As the project evolved, researchers became better at accommodating PPI and identifying PPI opportunities. It was generally agreed that PPI had a positive impact on the project overall, particularly on public engagement with the research. We concluded that the inclusion of both psychosocial and laboratory-based research in the same project facilitated PPI across all aspects of the research. In future projects, we would try to specify individual PPI activities in more detail at the project-planning stage, and better accommodate patient partners who are not native speakers of English.

**Abstract:**

**Background**

Patient and public involvement (PPI) enhances research quality and relevance and is central to contemporary health policy. The value of PPI has been recognised in rheumatology research, though there are limited examples of PPI in basic and translational science. The EU FP7 funded ‘EuroTEAM’ (**T**owards **E**arly biomarkers in **A**rthritis **M**anagement) project was established to develop biomarker-based approaches to predict the future development of rheumatoid arthritis and incorporated psychosocial research to investigate the perceptions of ‘at risk’ individuals about predictive testing, and to develop informational resources about rheumatoid arthritis (RA) risk. Patient involvement was central to EuroTEAM from the inception of the project. The objective of this paper is to describe the development of PPI in EuroTEAM, formatively assess the impact of PPI from the perspectives of researchers and patient research partners (PRPs), reflect on successes and lessons learned, and formulate recommendations to guide future projects.

**Methods**

Two mixed-methods surveys (for PRPs and researchers) and a teleconference were undertaken to assess the impact of PPI on individual work packages and on EuroTEAM overall.

**Results**

There was consensus about the positive impact of PPI on the research and on the experiences of those involved. In particular, the positive impact of PPI on the personal development of researchers, and on effective public engagement with EuroTEAM research were highlighted. Researchers described adapting their practice in future projects to facilitate PPI. Spin-off projects and ongoing collaborations between PRPs and researchers reflected the value of PPI to participants. PPI was more frequently integrated in psychosocial research, though examples of PPI in laboratory/translational science were also described. PRPs asked for more opportunities to contribute meaningfully to basic scientific research and for more extensive feedback on their contributions.

**Conclusions**

The findings were used to formulate recommendations to guide effective involvement of patients in future similar projects, including identifying specific training requirements for PRPs and researchers, the identification of PRP focused tasks/deliverables at the project planning stage, and supporting access to involvement for all PRPs. Importantly, the distinctive multidisciplinary approach of EuroTEAM, incorporating both basic science and psychosocial research, facilitated patient involvement in the project overall.

## Background

Patient-centred approaches are integral to contemporary health policy. Consequently, patient and public involvement (PPI) have become increasingly important in health research and service evaluation [1]. PPI has been defined as research being carried out “‘with’ or ‘by’ members of the public rather than ‘to’, ‘about’ or ‘for’ them” [2]. It is a central theme of the National Institute for Health Research (NIHR) in the United Kingdom, and evidence of active engagement with PPI is now a requirement for many funding bodies, including the European Union’s Horizon 2020 (H2020) funding stream. H2020 guidelines state that “Patients and citizens must be involved in the entire process, from the design of research programmes to the dissemination and implementation of results relating to wellbeing, health, social care, public health, and society” [3].

The involvement of patients in decisions that ultimately affect them has been accepted as beneficial to health research in a number of ways [4]. PPI enhances research quality, efficiency, and transparency, and helps ensure that public benefit is the key focus of the work undertaken. Consultation with those affected by research outcomes has augmented research relevance and validity, and facilitated wider dissemination and engagement with research from patients / members of the public. Patients have become involved with delivering research goals in several ways, at all stages of the research process. For example, they have contributed to priority setting, supported grant applications, advised on clinical trial design, provided feedback on patient facing documents, prepared lay summaries and supported other forms of research dissemination. Patients’ experience of living with a disease has brought additional and unique perspectives [5].

There has been a growing appreciation of the benefits of PPI within rheumatology research [6, 7]. For example, patients have contributed to: the development of questionnaires to assess help-seeking behaviours in patients with new onset rheumatoid arthritis (RA) [8] and hypothetical reactions to the symptoms of RA amongst members of the public [9]; the development of interview schedules [10–12]; systematic reviews [13–15]; generating research ideas [16]; rheumatology conferences [17] and the development of international research recommendations [18–20].

Effective PPI has fostered the development of ongoing, mutually advantageous relationships between researchers and patient research partners (PRPs) [21, 22]. However, maximising achievement of PPI goals has considerable resource implications. Funding may be required to compensate patients for their time and expenses, provide experienced staff to coordinate PPI and deliver appropriate training for researchers and PRPs. Researchers need to invest time into the development of resources to support meaningful patient involvement, such as lay summaries, glossaries and background information on key project elements [23]. Without resource allocation to support PPI, patient involvement risks becoming tokenistic [24, 25].

Whilst PPI has become increasingly commonplace in clinical and health services research, it has been less frequently integrated with early phase translational and laboratory based research [26, 27]. Although patients and researchers agree on the benefits of PPI in pre-clinical translational research in the context of rheumatology, associated challenges include communication between researchers and patients about scientific concepts and terminology and limited access to and awareness of PPI opportunities [28–30].

A further challenge associated with PPI is the difficulty in evaluating its impact [23]. Whilst there have been calls for more robust quantitative assessment of the impact of PPI on research quality, it has been argued that it is also important to capture the experiential, contextual impact of PPI on both researchers and patient research partners [31].

This paper describes the development and formative evaluation of the impact of PPI in a European Union FP7 funded translational research project, EuroTEAM - Towards Early biomarkers in Arthritis Management, which ran from 2012 to 2016. The aim of this multidisciplinary project was to develop new biomarker-based approaches to predict the development of RA in people who do not yet have the disease. RA is a chronic inflammatory disease affecting the joints and internal organs, and is associated with a reduced life expectancy of around 10 years. Early treatment of RA is associated with better clinical outcomes. If uncontrolled, this painful condition can lead to irreversible joint erosion and disability [32]. Improved prediction of RA development would facilitate early treatment and the development of preventive interventions.

An innovative aspect of the EuroTEAM project was that it encompassed both laboratory based science that addressed the identification of predictive biomarkers [33**–**39] and psychosocial research, that explored the perspectives of potential end-users of predictive tests for RA, and how disease related risk information could be communicated effectively to those at risk of developing RA in the future [40**–**44]. In order to ensure the quality and relevance of the research and to facilitate effective translation and dissemination of the research findings, PPI was considered an essential part of EuroTEAM from its inception. PPI was specifically funded and supported by the management team.

The objective of this paper is to describe the development of PPI in the EuroTEAM project, and the results of two mixed-methods surveys, undertaken towards the end of the project to capture the perspectives of both researchers and patients on the impact of PPI on this research. Our secondary objective is to reflect on the findings of this evaluation to identify good practice and areas for improvement to inform effective PPI in future projects of this kind.

## Methods

### Patient research partner involvement and EuroTEAM

The EuroTEAM project was a multidisciplinary collaboration between 16 partners across Europe [Table 1a]. The consortium comprised of 13 universities and 3 small/medium enterprises (SMEs) led by the University of Birmingham. EuroTEAM contained 5 work packages (WPs): genetic biomarkers (WP1), blood biomarkers (WP2), tissue biomarkers (WP3), dissemination and user integration (WP4), and project management (WP5). WPs 1–3 comprised of laboratory-based studies to identify biomarkers predictive of future RA development. The focus of WP4 was psychosocial research to elucidate the perspectives of individuals with an elevated risk of developing RA in the future (first degree relatives of existing RA patients and individuals with clinically suspect joint symptoms) [45]. The aim was to capture this group’s thoughts about being ‘at risk’, their acceptance of predictive testing and preferences for risk communication. A further objective of WP4 was the development and evaluation of informational material for individuals at risk of developing RA.

**Table 1:** Geographical distribution of EuroTEAM a) consortium partners and b) patient research partners (PRPs)

Country	Number of organizations
1a) EuroTEAM consortium partner organizations:	
Austria (Medical University of Vienna)	1
Germany (Charité, Universitätsklinikum Erlangen, Orgentec)	3
Iceland (deCODE)	1
The Netherlands (LUMC, AMC, Arthrogen)	3
Sweden (Karolinska Institutet, KTH, Uppsala Universitet)	3
Switzerland (University of Zurich)	1
United Kingdom (University of Birmingham, University of Leeds, University of Glasgow, University of Manchester)	4
Country	Number of PRPs
1b) EuroTEAM PRPs:	
Estonia	1
Germany	1
The Netherlands	1
Romania	2
Sweden	1
United Kingdom	3

During the early planning stages of EuroTEAM, several national RA organisations and patient groups in a range of European countries [Table 2] were approached in order to seek endorsement of the project, provide advice and support for the funding application, and to identify potential PRPs. Additional PRPs were recruited via clinician researchers and PRPs’ own networks, resulting in a panel of 9 PRPs; 8 patients with a diagnosis of RA and 1 person whose first-degree relative had RA [Table 1b]. PRP activity was co-ordinated by a research group manager and PRPs were compensated for travel/subsistence expenses incurred.

**Table 2:** European patient organisations approached to identify potential patient research partners

Organisation	Website
Swedish Rheumatism Association	www.reumatikerforbundet.org
National Rheumatoid Arthritis Society (UK)	www.nras.org.uk
Deutsche Rheuma-Liga	www.rheuma-liga-berlin.de
Rheuma Nederland (Netherlands)	https://reumanederland.nl/
EULAR - PARE (European League Against Rheumatism - People with Arthritis / Rheumatism across Europe)	www.eular.org/pare.cfm

PRPs contributed to a number of research activities over the 4 year project, with participation being voluntary at all times. Research activities with active patient partner involvement included:

- Attending and contributing to annual scientific meetings and regular teleconferences
- Development of a glossary resource (WP1–4)
- Contributing to a meta-synthesis of qualitative literature on public perceptions of predictive testing [13, 14] (WP4)
- Contributing to the development of interview schedules (including question setting) and the interpretation of qualitative data [12, 46] (WP4)
- Contributing to the development of informational resources for those at risk (WP4)
- Evaluating a web-based platform for the communication of risk information (WP4)
- Developing a questionnaire for patients undergoing a lymph node biopsy procedure (WP3)
- Developing informational resources for patients about RA such as the ‘Metaphor Project’ (a collaboration between Eva C Johansson (PRP) and Dr. Heidi Wähämaa from the Karolinska Institutet) exploring the communication of scientific concepts via the use of metaphors and visual representations
- Contributing to the project website (e.g. providing news items and reports, creating subtitles for videos) (WP4)
- Developing lay summaries of EuroTEAM methods and findings (e.g. lay summary of metabolomics in EuroTEAM) (WP2)
- Developing posters for dissemination at the European League Against Rheumatism (EULAR) Congress (WP1–4)

Training for PRPs was provided on an ad-hoc basis for specific research activities (e.g. analysis and interpretation of qualitative data) [14]. Annual project meetings included a day focused specifically on PRPs, where presentations were delivered by the researchers in lay language. No formal training for researchers was provided, however EULAR patient involvement reference cards were utilised by some consortium members [47]. Many researchers adapted to accommodate PRP needs as the project evolved, due to increased mutual understanding. For example, lay summaries were incorporated into research presentations, and frequent short breaks were introduced at annual meetings to avoid PRPs sitting for long periods. Reporting of patient involvement is summarized in Additional file 3.

### Evaluation of PPI in EuroTEAM

Towards the end of the EuroTEAM project, two surveys (one for PRPs and another for researchers; Additional files 1 and 2, respectively) were developed to evaluate the impact of PPI in EuroTEAM. The surveys included Likert-type response scales and free text responses and asked respondents to evaluate the impact of PPI in WPs1–3 and WP4 separately, as well as the impact of PPI on the project as a whole. The design and content of the surveys themselves were developed in collaboration with PRPs to ensure they were understandable and comprehensive. The surveys were distributed in April 2016 via email to all 9 PRPs and 51 researchers [Table 3]. Email reminders were sent out to non-responders after 2 weeks. Responses were collated and summarised descriptively. The frequency with which each response category was chosen was summed for Likert-type scales, and descriptive thematic analysis of free text responses was undertaken by RB and MF. A teleconference was held with PRPs and researchers to review and interpret the survey findings and their implications for future projects. Two PRPs also contributed to the preparation of this manuscript.

**Table 3:** Survey recipients

Country	Number of people who received the survey
3a) EuroTEAM researchers:	
Austria (Medical University of Vienna)	4
Germany (Charité, Universitätsklinikum Erlangen, Orgentec)	7
Iceland (deCODE)	1
The Netherlands (LUMC, AMC, Arthrogen)	8
Sweden (Karolinska Institutet, KTH, Uppsala Universitet)	7
Switzerland (University of Zurich)	4
United Kingdom (University of Birmingham, University of Leeds, University of Glasgow, University of Manchester)	20
3b) EuroTEAM PRPs:	
Estonia	1
Germany	1
The Netherlands	1
Romania	2
Sweden	1
United Kingdom	3

## Results

### Respondents

Sixty-six per cent (6 out of 9) of PRPs and 29% (15 out of 51) of researchers completed the surveys. All the PRPs who responded were female. Two were aged between 30 and 39 years, two aged 50–59 years and two were aged between 60 and 69 years.

Eleven of the 15 researchers were female. Four were aged between 30 and 39 years, eight were aged 40–49 years, two were 50–59 years and one was aged between 60 and 69 years). Fourteen were from academic institutions, one was from an SME. Six were clinical researchers, nine were non-clinical researchers.

### Survey for patient research partners

The quantitative results of the survey for PRPs are summarized in Table 4. All of the PRP respondents reported that their involvement had a positive overall impact on the EuroTEAM project. This was mainly described in terms of the effect of their involvement on researchers’ perspectives and ability to communicate with members of the public:

*“A present, physical reminder of the lay aspects of communication and the patient perspective.”*

Respondents stated they had made a large or moderate positive contribution to WP4, and described being involved in specific research activities:

*“Contribution at coding and summarizing activity, study protocol, leaflet/brochures for patients, dissemination (in the future), feedback questionnaires, video transcript, writing an article in PARE [People with Arthritis/Rheumatism in Europe)] e-Breakthrough, EuroTEAM website”*

The majority of the PRPs thought they had made a minor or moderate positive contribution to WPs 1–3, with one PRP reporting a larger contribution

*“I think we made a large contribution to the lymph node biopsy questionnaire for WP3, but for WP1 and WP2 maybe not so much, apart from the overall impact.”*

Most of the PRPs found they had a moderate understanding of EuroTEAM from the outset. Half of the PRPs said there were about the right number of assignments for them, whereas half stated that there were too few assignments. All agreed that the assignments were of an appropriate level of difficulty and that they received suitable levels of information about them, however some respondents mentioned that more contextual information or specific training and feedback would have been useful. Half of the respondents stated that they had received too little feedback on the outcome of their contributions to this project:

*“When introducing a completely new skill – e.g. coding – perhaps more training, and feedback, would be helpful”*

All respondents agreed that the glossary resource was either very useful or extremely useful, and that PRP involvement in EuroTEAM was well co-ordinated. PRP contributions were reported to have been welcomed by researchers and fairly acknowledged:

*“We were welcomed and included in the team. We were listened to with respect. We were included in setting recommendations for future work.”*

Most PRPs mentioned that their contribution to meetings was very or extremely well supported, although one reported that there had been a moderate amount of support. Some described how this support evolved over the project:

*“By the end of the project, with PRPs in the room, the researchers automatically featured some lay language in their presentations, avoiding the need for separate briefing: by this stage the PRPs themselves were also more familiar with the scientific concepts and language of the project. So we all learnt together.”*

For many respondents, building relationships with researchers was one of the most valuable aspects of participating in EuroTEAM:

*“Enjoyed opportunity to talk to researchers and especially encouraging young researchers to interact with patients.”*

In particular, PRPs valued being treated as respected equal partners in the research process …

*“Never felt patronised but always valued.”*…

and found that their involvement with EuroTEAM had had a positive impact on them personally:

*“A definite positive impact. Very informative and challenging in a good way. I’ve definitely grown as a person through the work and meeting all the people in the consortium”.*

Such interactions between researchers and PRPs also led to the development of innovative spin-off collaborations:

*“The Metaphor project showed pure imagination.”*

All respondents to the survey for PRPs were either very interested or extremely interested in contributing to future projects as a PRP. When asked how PRP involvement could have been improved in EuroTEAM, some described accessibility issues relating to accommodation provided at annual project meetings, and several made suggestions to support communication between researchers and PRPs, such as: the need for short breaks during meetings; more materials in lay language; more feedback on project progress and the outcome of PRPs contributions; lay summaries and printouts of slides used in presentations; consideration of non-native English speakers’ needs during teleconferences and face to face meetings.

**Table 4:** Quantitative results of the survey for patient research partners (PRPs; *N* = 6)

Q1. What kind of impact has PRP involvement had on EuroTEAM overall?							
	Extremely negative impact	Negative impact	Slightly negative impact	No impact	Slightly positive impact	Positive impact	Extremely positive impact
Number (%) of PRPs	0	0	0	0	0	6 (100%)	0
Q2. How much do you feel you have been able to contribute positively to WPs 1–3? (WP1 genetics, WP2 blood, WP3 tissue)							
	No contribution at all	Minor contribution	Moderate contribution	Large contribution	Extremely large contribution		
Number (%) of PRPs	0	3 (50%)	2 (33%)	1 (17%)	0		
Q3. How much do you feel you have been able to contribute positively to WP4 (dissemination & user integration)?							
	No contribution at all	Minor contribution	Moderate contribution	Large contribution	Extremely large contribution		
Number (%) of PRPs	0	0	3 (50%)	3 (50%)	0		
Q4. How well did you feel you understood the objectives, methods and outcomes of EuroTEAM?							
	Did not understand at all	Understood a little	Moderate understanding	Understood a lot	Understood everything		
Number (%) of PRPs	0	0	5 (83%)	1 (17%)	0		
Q5. How did you feel about the number of assignments for PRPs in EuroTEAM?							
	Far too few assignments	Too few assignments	About the right number of assignments	Too many assignments	Far too many assignments		
Number (%) of PRPs	0	3 (50%)	3 (50%)	0	0		
Q6. How did you feel about the level of difficulty of the assignments for PRPs in EuroTEAM?							
	Far too difficult	Too difficult	About the right level of difficulty	Too easy	Far too easy		
Number (%) of PRPs	0	0	6 (100%)	0	0		
Q7. How did you feel about the amount of information you received about assignments for PRPs in EuroTEAM?							
	Far too little information	Too little information	About the right amount of information	Too much information	Far too much information		
Number (%) of PRPs	0	0	6 (100%)	0	0		
Q8. How useful did you find the glossary for PRPs?							
	Not at all useful	Not very useful	Moderately useful	Very useful	Extremely useful		
Number (%) of PRPs	0	0	0	5 (83%)	1 (17%)		
Q9. How welcome did you feel your opinions were?							
	Not at all welcome	Not very welcome	Moderately welcome	Very welcome	Extremely welcome		
Number (%) of PRPs	0	0	0	4 (67%)	2 (33%)		
Q10. How well was PRP involvement co-ordinated?							
	Not at all well- coordinated	Not very well- coordinated	Moderately well-coordinated	Very well- coordinated	Extremely well-coordinated		
Number (%) of PRPs	0	0	0	1 (17%)	5 (83%)		
Q11. How did you feel about the amount of feedback you received on the outcome of your contribution to assignments for PRPs?							
	Far too little feedback	Too little feedback	About the right amount of feedback	Too much feedback	Far too much feedback		
Number (%) of PRPs	1 (17%)	2 (33%)	3 (50%)	0	0		
Q12. How did you feel about how fairly your contributions to the EuroTEAM project have been acknowledged?							
	Not at all fairly	Not very fairly	Moderately fairly	Very fairly	Extremely fairly		
Number (%) of PRPs	0	0	0	3 (50%)	3 (50%)		
Q13. How well did you feel your contribution to EuroTEAM meetings was supported?							
	Not at all well supported	Not very well supported	Moderately well supported	Very well supported	Extremely well supported		
Number (%) of PRPs	0	0	1 (17%)	2 (33%)	3 (50%)		
Q14. How interested are you in contributing to future research projects as a PRP?							
	Not at all interested	Not very interested	Moderately interested	Very interested	Extremely interested		
Number (%) of PRPs	0	0	0	2 (33%)	4 (67%)		

Some also identified missed opportunities for dissemination:

*“I think the PRPs themselves could have written (or should write) an abstract about their experiences and submit it for the PARE sessions at the EULAR congress.”*

### Survey for researchers

The quantitative results of the survey for researchers are summarised in Table 5. Respondents had varying levels of previous experience of working with PRPs, ranging from none to extensive experience, however the majority of researchers reported no prior experience. All respondents were in agreement that PRP involvement had a positive impact on EuroTEAM:

*“The patient research partners have not only given an insight into what it actually entails to have RA and which aspects they feel we need to address in our research, they have contributed enormously to the project, both intellectually and practically. It has made the project’s outcomes more valuable to the RA community, relatives and friends as well as the research community.”* (non-clinical researcher, academic institution)

*“The respect which was shown for PRPs (for example, mini pauses) created an overall kind and positive atmosphere. PRP presence reminds us why we do research. For non-clinical scientists it is really important to meet patients – for some scientists EuroTEAM might have been the first time?”* (non-clinical researcher, academic institution)

Several researchers recounted that they had learnt a great deal from their involvement with PRPs, particularly in relation to their ability to engage with the public about their research:

“*I personally learned a lot about how to translate scientific terminology into understandable language to meet the demands of a lay audience. It was advantageous for the project that the patient focus sometimes differed from the scientific/medical focus.”* (communications manager, SME)

Of the five respondents who were most involved in WP2, one mentioned that PRPs had not been able to contribute positively to this work package:

*“The PRPs were very interested in our research, but did not/could not contribute to the research questions, methods or interpretation of the results.”* (clinical researcher, academic institution)

Another said that PRPs had been able to make a minor contribution, whereas another stated that PRPs had contributed moderately and two found that PRPs had been able to make a large contribution to this work package.

**Table 5:** Quantitative results of the survey for researchers (*N* = 15)

Q1. How much experience of working with PRPs did you have before your involvement with EuroTEAM?							
	No experience at all	Slight experience	Moderate experience	A good deal of experience	Extensive experience		
Number (%) of researchers	7 (46.7%)	2 (13.3%)	2 (13.3%)	2 (13.3%)	2 (13.3%)		
Q2a. Which WP have you been most involved with?							
	WP1 (genetic biomarkers)	WP2 (blood biomarkers)	WP3 (tissue biomarkers)	WP4 (user integration)			
Number (%) of researchers	0	5 (33.3%)	4 (26.7%)	6 (40.0%)			
Q2b. How much do you feel that PRPs have been able to contribute positively to this WP?							
	No contribution at all	Minor contribution	Moderate contribution	Large contribution	Extremely large contribution		
Number (%) of researchers	2 (13.3%)	2 (13.3%)	1 (6.7%)	7 (46.7%)	3 (20.0%)		
Related WP	WP2 (1) WP3 (1)	WP2 (1) WP3 (1)	WP2 (1)	WP2 (2) WP3 (2) WP4 (3)	WP4 (3)		
Q3a. Which other WPs have you been involved with (if any)?^1^							
	WP1 (genetic biomarkers)	WP2 (blood biomarkers)	WP3 (tissue biomarkers)	WP4 (user integration)			
Number (%) of researchers	2 (40%)	0 (0%)	2 (40%)	1 (20%)			
Q3b. How much do you feel that PRPs have been able to contribute positively to this WP?							
	No contribution at all	Minor contribution	Moderate contribution	Large contribution	Extremely large contribution		
Number (%) of researchers	2 (40%)	1 (20%)	1 (20%)	0 (0%)	1 (20%)		
Related WP	WP1 (2)	WP3 (1)	WP3 (1)	N/A	WP4 (1)		
Q4. What kind of impact do you think PRP involvement has had on EuroTEAM overall?							
	Extremely negative impact	Negative impact	Slightly negative impact	No impact	Slightly positive impact	Positive impact	Extremely positive impact
Number (%) of researchers	0	0	0	0	2 (13.3%)	6 (40%)	7 (46.7%)
Q5. Did you have any practical experience of working with patients within EuroTEAM?							
	Yes	No					
Number (%) of researchers	10 (66.7%)	5 (33.3%)					

^1^5 *of the 15 respondents were involved with more than one work package*

Of the four respondents who were most involved in WP3, one found that the PRPs had not been able to contribute positively, others stated that PRPs had made a minor (*N* = 1) or large (*N* = 2) contribution to this work package.

Amongst the six respondents who were most involved in WP4, there was agreement that PRPs had been able to make a large (*N* = 3) or extremely large (*N* = 3) contribution:

*“Their contribution has been very substantial … They have been involved with the coding and analysis of interviews. They have been an integral part of the development of the resources for people at risk of RA. PRPs have been instrumental in the development of the information resources we developed for people at risk of RA.”* (non-clinical researcher, academic institution)

Five of the 15 researchers had been involved in more than one work package. Two of these had additionally been involved in WP1, and reported that PRPs had not been able to make a positive contribution at all, two had also been involved in WP3 and reported that PRPs had made a minor or moderate contribution. One researcher had additional involvement in WP4 and reported that PRPs had made an extremely large contribution to that work package.

The majority of PRP involvement was perceived to be focused around the psychosocial research carried out in WP4 and researchers sometimes found it difficult to identify meaningful ways to involve patients in other work packages:

*“All work packages and research projects should have had input from PRPs and perhaps this should have been implemented earlier on. It should be an integral part of all the research carried out from the very beginning. In some of the more basic research this might be difficult on a practical level, but lay summaries of the findings at various stages are a minimum.”* (non-clinical researcher, academic institution)

Those who actively involved PRPs found it very rewarding. For example:

*“I had direct talks with patients followed by email correspondence in developing lay summaries of technologies. These were overwhelmingly positive, with positive involvement of the patients and a good and worthwhile contribution to the refinement of informational sources.”* (non-clinical researcher, academic institution)

*“Close involvement with the partners with producing lay summary of metabolomics technologies to augment a broader understanding.”* (non-clinical researcher, academic institution)

When asked how PRP involvement in EuroTEAM had affected how they would involve PRPs in future projects, several researchers indicated that their perception of PPI had evolved throughout the project:

*“Before working on EuroTEAM I was always somewhat wary of the idea of working with patients on research – I worried that they wouldn’t understand our work and that their direction would move research away from basic science to outcomes that aren’t the sort of thing I can do. However, I was mistaken. They are brilliant, enthusiastic and very supportive!”* (non-clinical researcher, academic institution)

In addition, it was mentioned that PRPs could have a greater role in setting the research agenda:

*“It would have been interesting to hear what individual PRPs think about the ongoing research and get their input on which kind of research they think we should prioritise and focus on. This would increase our insights (especially non-clinical researchers) what RA patients experience as a big health issues affecting their daily life.” (non-clinical researcher, academic institution)*

Others described gaining insight that would change their approach in future projects, and highlighted that they would endeavour to find more meaningful ways for PRPs to be involved in basic scientific research:

*“I would be more proactive in identifying tasks for PRPs to get involved with in the context of biological research studies to allow for their better integration into these projects.”* (clinical researcher, academic institution)

*“Try to find ways of asking for very specific, as well as general feedback on research.”* (non-clinical researcher, academic institution)

It was also suggested that facilitating small-scale meetings alongside annual project meetings might facilitate communication between researchers and PRPs:

*“Discussions on a personal level with PRPs present at the annual meeting happened during coffee/lunch breaks or dinner. Sometimes it was a bit hard to start a conversation and for the junior researchers it might not always have been clear, who the PRPs were and what they could talk about. Maybe this could be more guided somehow. Maybe by introducing a kind of speed date session for researchers and PRPs at the start of the project? Guided by the EULAR reference cards?”* (non-clinical researcher, academic institution)

Some researchers believed that PPI should be incorporated into basic training for all researchers:

*“I think that PRPs could (should?) be included as part of the PhD education:*

- *To practice how to communicate with lay people*
- *To meet a “real patient”, not only working with patient samples”*

(non-clinical researcher, academic institution)

Some researchers mentioned that as a direct result of their positive experiences of working with PRPs in EuroTEAM, they had gone on to include PRPs in other projects:

*“From EuroTEAM we recruited our “own” PRP to our research group … Overall, she has been a very important link between our rheumatologists and non-clinical PhD students and post-docs... Our group meetings are not only about cells and molecules anymore, we have also patient-orientated discussions with our rheumatologists.”* (non-clinical researcher, academic institution)

*“The involvement of PRPs was very important for my personal development. In the past I had no idea on how to contact them (as a non-clinical researcher) and how we could together bring research to a higher level. For me a new door has opened, and currently I am in contact with PRPs to discuss my future research proposal.”* (non-clinical researcher, academic institution)

## Discussion

These findings demonstrate the positive impact of PPI on a multi-centre, multidisciplinary European translational research project that incorporated both laboratory/translational science and psychosocial research. PRPs and researchers who completed the surveys reported that the involvement of PRPs in the project enhanced the research and their own experience of participating in this project. Both groups described the value of building ongoing relationships between patients and researchers and interactively developing their own knowledge and communication skills in ways that were not only personally rewarding but also facilitated effective public engagement and dissemination of the research findings. The development of innovative spin-off projects and ongoing future collaborations between PRPs and researchers reflected the value of PPI to those involved.

There was agreement between all respondents that the PRPs were able to make significant positive contributions to the psychosocial research and dissemination activities associated with WP4, but several reported that PRPs were less able to make a meaningful contribution to the laboratory-based research undertaken in WPs1–3. However, there were examples of effective patient contributions to these work packages that were valued by researchers and PRPs, such as the development of a questionnaire for patients undergoing a lymph node biopsy procedure, lay summaries of metabolomics studies, and lay posters of EuroTEAM findings, which were presented at the European League against Rheumatism (EULAR) Annual Meeting 2016. Several researchers mentioned that in future, they would actively try to identify specific (rather than general) ways in which patients could contribute to laboratory-based projects and dissemination of project results. It is possible that laboratory-based researchers’ awareness of PPI and confidence to implement it in their own research increased in response to proximity with the PPI activity that developed in WP4. In this respect, the distinctive multidisciplinary structure of EuroTEAM facilitated the evolution of PPI in this project. Moving towards more integrative, interdisciplinary models may further enhance PPI by developing common ground that is conducive to collaboration between patients and researchers.

The challenges associated with the involvement of patients in early stage translational science could be at least partly be addressed by appropriate training for researchers about the benefits of PPI and how to identify opportunities and overcome barriers to useful PRP involvement. This could be further supported at the project planning stage by incorporation of deliverables and/or milestones that specifically relate to activities involving PRPs, such as the development of lay progress reports. However, it may be difficult to specify in advance the extent to which patients are able or willing to be involved. It is important to avoid imposing heavy workloads or tight deadlines on patients who may have limited availability and are continuously managing the impact of a chronic condition on their lives, which may also be busy with work, family or other commitments. It is important that patients do not feel pressurised to contribute to research activities unwillingly in order to satisfy research objectives or funders’ requirements.

A further issue raised was that PRPs thought that they would have benefitted from more direct feedback on the outcome or impact of their contributions to research activities, and ongoing progress of research studies within the project. Researchers may not always have been aware of the extent to which feedback could be provided to PRPs, or have had the time needed to provide such feedback. These findings align with recent work to clarify the kinds of feedback needed by public contributors to research [48]. These issues could also be addressed, in collaboration with PRPs, at the project planning stage. Training may be required (for both researchers and PRPs) and resources allocated to support the development of effective PPI, especially where local or institutional infrastructure or funding to support such activities is insufficient or absent. It was suggested in the current evaluation and elsewhere [28] that PPI related training could be integrated into basic training for research students. It is not difficult to imagine that funding bodies will increasingly expect institutional infrastructure, as well as project-specific capacity to support such PPI facilitators to be demonstrated, to ensure research relevance and impact.

### Strengths and limitations

A strength of the surveys described here was the use of both quantitative and qualitative methods, which facilitated formative empirical assessment of the impact of PPI on the EuroTEAM project, and captured the experiential and contextual impact from the perspectives of the researchers and PRPs involved. The impact of PPI can be framed in terms of impact on the research itself or impact on the stakeholders involved [31, 49]. For example, some of the researchers in the present survey described how they had gained knowledge about RA from the patients’ expertise, and how they would alter their practice to facilitate PPI and dissemination in future projects as a direct result of their participation in EuroTEAM.

A second strength of the surveys was that they were developed in collaboration with PRPs, to ensure that the questions included were comprehensive from their perspective. PRPs also contributed to the interpretation of the survey results and the preparation of this manuscript, ensuring that their viewpoints were fully and fairly represented. A further strength was the pan-European nature of both PRPs and researchers.

A limitation of the surveys was that the respondents are unlikely to be representative. PRPs were mostly active members of patient organisations and therefore fully engaged with the promotion of patient representation and involvement. However, this can be said of most patients who are actively involved in research and is not specific to this study. Not all PRPs/researchers responded to the survey, and the response rate was particularly low amongst researchers, despite email reminders. With low participant numbers, the utility of the quantitative approach beyond formative evaluation was limited. Furthermore, as surveys were returned via email, respondents were identifiable. It is therefore possible that the results were susceptible to social desirability bias, or that those who responded were more likely to be proactively engaged with PPI and favourable towards the involvement of PRPs in a project of this kind.

The notion of representativeness of PRPs is a matter of ongoing debate [50]. Self-selected patient representatives often develop considerable research expertise in their own right, and this empowers them to contribute meaningfully to the research process. However, this could also be considered to detract from their position as representatives of the experience of lay people [28]. Ensuring that patients from all social groups have equal access to PPI is challenging, and the ability of patients at different stages of a disease to participate is likely to be variable, though they may have very different perspectives on research priorities.

We conducted this formative evaluation of PPI towards the end of the project, but note that PPI practice adapted throughout in response to PRPs needs and feedback on specific elements, such as annual scientific meetings. More formal, iterative assessment of PRP satisfaction throughout the project lifecycle could have supported this adaptive process [28].

## Conclusions and recommendations

There was consensus among PRPs and researchers about the positive impact of PPI on EuroTEAM. These findings suggest that PPI can be usefully incorporated in future projects of this kind, and that barriers and facilitators to the implementation of effective PPI in this context should be identified and addressed. On the basis of these findings, it is possible to make the following recommendations, which we hope will guide future projects:

- Initiatives to support communication between researchers and patients, such as glossaries and advance lay summaries of research presentations were highly valued and could usefully be incorporated and further developed in future projects.
- The unusual multidisciplinary structure of EuroTEAM facilitated patient involvement in the project overall. Further research is needed to define the ways in which multi- and interdisciplinary research can enhance PPI.
- Additional training would have been useful for both researchers and PRPs to support effective PPI and the development of mutually advantageous relationships. For example, identification of opportunities for meaningful PPI, awareness of PRP needs, providing feedback and developing communication skills.
- It would have been useful to identify specific tasks/areas for development with PRPs at the project planning stage and to consider including such activities as deliverables/milestones where feasible.
- Regular opportunities for feedback on the impact of PRPs contribution to specific research activities, and on the progress of the project overall, was highly valued, and could also have been specified at the project planning stage.
- Equality of access to patient representation could have been addressed more effectively, both when planning PRP recruitment and throughout the project; for example by facilitating awareness and access to PPI to all patients, not just those who are active members of patient organisations; providing translation services and support for non-native speakers; and supporting research involvement for individuals with particular literacy or mobility/access needs.

## Supplementary information

**Additional file 1.** Survey for patient research partners (PRPs).

**Additional file 2.** Survey for researchers.

**Additional file 3.** GRIPP 2.

## Data Availability

The data collected during the evaluation exercise described in this paper are available from the corresponding author on reasonable request.

## Abbreviations

EULAR: European League Against Rheumatism
EuroTEAM: EU FP7 funded collaborative project – Towards Early biomarkers in Early Arthritis
H2020: Horizon 2020 (EU funding stream)
NIHR: National Institute for Health Research
PARE: People with Arthritis / Rheumatism in Europe
PPI: Patient and public involvement
PRP: Patient research partner
RA: Rheumatoid arthritis
SMEs: Small / medium enterprises
WP: Work package
