# Supplementary material for: Development and formative evaluation of patient research partner involvement in a multi-disciplinary European translational research project
Source: Res Involv Engagem. 2020 Feb 19;6:6. doi: 10.1186/s40900-020-0178-7 (PMC7031919; doi:10.1186/s40900-020-0178-7)
Supplement: Supplementary file 1 — Additional file 1. Survey for patient research partners (PRPs). [file 40900_2020_178_MOESM1_ESM.docx]

**Supporting Information 1. Survey for patient research partners (PRPs)**

We would like to capture your thoughts about Patient Research Partner involvement with the EuroTEAM project over its 4 year duration. In particular we are keen to understand what went well and where we could improve.

| 1. **Please tick a box to indicate your age (in years):**  \| **🗆** \| **🗆** \| **🗆** \| **🗆** \| **🗆** \| **🗆** \| \| --- \| --- \| --- \| --- \| --- \| --- \| \| 18-29 \| 30-39 \| 40-49 \| 50-59 \| 60-69 \| 70+ \| |
| --- | --- | --- | --- | --- | --- | --- | --- | --- | --- | --- | --- | --- |
| 1. **Please tick a box to indicate your gender:**  \| **🗆** \| **🗆** \| \| --- \| --- \| \| Female \| Male \| |
| 1. **Please tick a box to show what kind of impact you think Patient Research Partner involvement has had on EuroTEAM overall:**  \| **🗆** \| **🗆** \| **🗆** \| **🗆** \| **🗆** \| **🗆** \| **🗆** \| \| --- \| --- \| --- \| --- \| --- \| --- \| --- \| \| Extremely negative impact \| Negative impact \| Slightly negative impact \| No impact \| Slightly positive impact \| Positive impact \| Extremely positive impact \| |
| **Please describe any impact you think that Patient Research Partner involvement has had on EuroTEAM overall:** |
| 1. **Please tick a box to show how much you feel you have been able to contribute positively to Work Packages 1 – 3 (genetic, blood and tissue based biomarker related work packages):**  \| **🗆** \| **🗆** \| **🗆** \| **🗆** \| **🗆** \| \| --- \| --- \| --- \| --- \| --- \| \| No contribution at all \| Minor contribution \| Moderate contribution \| Large contribution \| Extremely large contribution \| |
| **Please describe how, if at all, you feel you have been able to contribute positively to Work Packages 1-3:** |
| 1. **Please tick a box to show how much you feel you have been able to contribute positively to Work Package 4 (dissemination and patient and relative perspectives on risk related work package):**  \| **🗆** \| **🗆** \| **🗆** \| **🗆** \| **🗆** \| \| --- \| --- \| --- \| --- \| --- \| \| No contribution at all \| Minor contribution \| Moderate contribution \| Large contribution \| Extremely large contribution \| |
| **Please describe how, it at all, you feel you have been able to contribute positively to Work Package 4:** |
|  |
| 1. **Please tick a box to show how well you feel you understood the objectives, methods and outcomes of EuroTEAM:**  \| **🗆** \| **🗆** \| **🗆** \| **🗆** \| **🗆** \| \| --- \| --- \| --- \| --- \| --- \| \| Did not understand at all \| Understood a little \| Moderate understanding \| Understood a lot \| Understood everything \| |
| 1. **Please tick a box to show how you feel about the number of assignments for patient research partners in EuroTEAM:**  \| **🗆** \| **🗆** \| **🗆** \| **🗆** \| **🗆** \| \| --- \| --- \| --- \| --- \| --- \| \| Far too few assignments \| Too few assignments \| About the right number of assignments \| Too many assignments \| Far too many assignments \| |
| 1. **Please tick a box to show how you feel about the level of difficulty of the assignments for patient research partners in EuroTEAM:**  \| **🗆** \| **🗆** \| **🗆** \| **🗆** \| **🗆** \| \| --- \| --- \| --- \| --- \| --- \| \| Far too difficult \| Too difficult \| About the right level of difficulty \| Too easy \| Far too easy \| |
| 1. **Please tick a box to show how you feel about the amount of information you received about the assignments for patient research partners in EuroTEAM:**  \| **🗆** \| **🗆** \| **🗆** \| **🗆** \| **🗆** \| \| --- \| --- \| --- \| --- \| --- \| \| Far too little information \| Too little information \| About the right amount of information \| Too much information \| Far too much information \| |
| 1. **Please tick a box to show how useful you found the EuroTEAM Glossary for Patient Research Partners:**  \| **🗆** \| **🗆** \| **🗆** \| **🗆** \| **🗆** \| \| --- \| --- \| --- \| --- \| --- \| \| Not at all useful \| Not very useful \| Moderately useful \| Very useful \| Extremely useful \| |
| 1. **Please tick a box to show how welcome you felt your opinions were:**  \| **🗆** \| **🗆** \| **🗆** \| **🗆** \| **🗆** \| \| --- \| --- \| --- \| --- \| --- \| \| Not at all welcome \| Not very welcome \| Moderately welcome \| Very welcome \| Extremely welcome \| |
| 1. **Please tick a box to show how well Patient Research Partner involvement was coordinated:**  \| **🗆** \| **🗆** \| **🗆** \| **🗆** \| **🗆** \| \| --- \| --- \| --- \| --- \| --- \| \| Not at all well coordinated \| Not very well coordinated \| Moderately well coordinated \| Very well coordinated \| Extremely well coordinated \| |
| 1. **Please tick a box to show how you feel about the amount of feedback you received on the outcome of your contribution to assignments for Patient Research Partners:**  \| **🗆** \| **🗆** \| **🗆** \| **🗆** \| **🗆** \| \| --- \| --- \| --- \| --- \| --- \| \| Far too little feedback \| Too little feedback \| About the right amount of feedback \| Too much feedback \| Far too much feedback \| |
| 1. **Please tick a box to show how you feel about how fairly your contributions to the EuroTEAM project have been acknowledged:**  \| **🗆** \| **🗆** \| **🗆** \| **🗆** \| **🗆** \| \| --- \| --- \| --- \| --- \| --- \| \| Not at all fairly \| Not very fairly \| Moderately fairly \| Very fairly \| Extremely fairly \| |
| 1. **Please tick a box to show how well you feel your contribution to EuroTEAM meetings was supported:**  \| **🗆** \| **🗆** \| **🗆** \| **🗆** \| **🗆** \| \| --- \| --- \| --- \| --- \| --- \| \| Not at all well supported \| Not well interested \| Moderately well supported \| Very well supported \| Extremely well supported \| |
| 1. **Please tick a box to show how interested you are in contributing to future research projects as a Patient Research Partner:**  \| **🗆** \| **🗆** \| **🗆** \| **🗆** \| **🗆** \| \| --- \| --- \| --- \| --- \| --- \| \| Not at all interested \| Not very interested \| Moderately interested \| Very interested \| Extremely interested \| |
| 1. **What went well in terms of Patient Research Partner involvement in EuroTEAM?** |
|  |
| 1. **Can you suggest ways that Patient Research Partner involvement in EuroTEAM could have been improved?** |
|  |
| 1. **Has your involvement with the EuroTEAM project had an impact on you, either positive or negative? If so, please describe the impact:** |
|  |

**Thank you!**
