# Supplementary material for: Development and formative evaluation of patient research partner involvement in a multi-disciplinary European translational research project
Source: Res Involv Engagem. 2020 Feb 19;6:6. doi: 10.1186/s40900-020-0178-7 (PMC7031919; doi:10.1186/s40900-020-0178-7)
Supplement: Supplementary file 2 — Additional file 2. Survey for researchers. [file 40900_2020_178_MOESM2_ESM.docx]

**Supporting Information 2. Survey for researchers**

We would like to capture your thoughts about Patient Research Partner involvement with the EuroTEAM project over its 4 year duration. In particular we are keen to understand what went well and where we could improve.

| 1. Please tick a box to show which type of organisation you represent:  \| **🗆** \| **🗆** \| \| --- \| --- \| \| Academic partner \| Industry partner \| |
| --- | --- | --- | --- | --- |
| 1. Please tick a box to indicate your position:  \| **🗆** \| **🗆** \| **🗆** \| \| --- \| --- \| --- \| \| Clinical researcher \| Non-clinical researcher \| Other \|   Please describe your position: |
| 1. Please tick a box to indicate your age (in years):  \| **🗆** \| **🗆** \| **🗆** \| **🗆** \| **🗆** \| **🗆** \| \| --- \| --- \| --- \| --- \| --- \| --- \| \| 18-29 \| 30-39 \| 40-49 \| 50-59 \| 60-69 \| 70+ \| |
| 1. Please tick a box to indicate your gender:  \| **🗆** \| **🗆** \| \| --- \| --- \| \| Female \| Male \| |
| 1. Please tick a box to show how much experience of working with Patient Research Partners you had before your involvement with EuroTEAM? |
| \| **🗆** \| **🗆** \| **🗆** \| **🗆** \| **🗆** \| \| --- \| --- \| --- \| --- \| --- \| \| No experience at all \| Slight experience \| Moderate experience \| A good deal of experience \| Extensive experience \| |
| Please describe any experience of working with Patient Research Partners you had before your involvement with EuroTEAM: |
| For the Work Packages that you have been most associated with, please tell us how much you feel that patient research partners have been able to contribute positively: |
| 6. Please tick a box to show which Work Package you have been most involved in:   \| **🗆** \| **🗆** \| **🗆** \| **🗆** \| \| --- \| --- \| --- \| --- \| \| WP1 \| WP2 \| WP3 \| WP4 \| |
| Please tick a box to show how much you feel that patient research partners have been able to contribute positively to this Work Package:   \| **🗆** \| **🗆** \| **🗆** \| **🗆** \| **🗆** \| \| --- \| --- \| --- \| --- \| --- \| \| No contribution at all \| Minor contribution \| Moderate contribution \| Large contribution \| Extremely large contribution \| |
| Please describe how, if at all, you feel that patient research partners have been able to contribute positively to this Work Package: |
| 7. Please tick a box to show which other Work Package you have been involved in (if any):   \| **🗆** \| **🗆** \| **🗆** \| **🗆** \| \| --- \| --- \| --- \| --- \| \| WP1 \| WP2 \| WP3 \| WP4 \| |
| Please tick a box to show how much you feel that patient research partners have been able to contribute positively to this Work Package:   \| **🗆** \| **🗆** \| **🗆** \| **🗆** \| **🗆** \| \| --- \| --- \| --- \| --- \| --- \| \| No contribution at all \| Minor contribution \| Moderate contribution \| Large contribution \| Extremely large contribution \| |
| Please describe how, if at all, you feel that patient research partners have been able to contribute positively to this Work Package: |
| 8. Please tick a box to show what kind of impact you think Patient Research Partner involvement has had on EuroTEAM overall:   \| **🗆** \| **🗆** \| **🗆** \| **🗆** \| **🗆** \| **🗆** \| **🗆** \| \| --- \| --- \| --- \| --- \| --- \| --- \| --- \| \| Extremely negative impact \| Negative impact \| Slightly negative impact \| No impact \| Slightly positive impact \| Positive impact \| Extremely positive  impact \| |
|  |
| Please describe any impact Patient Research Partner involvement has had on EuroTEAM overall: |
| 9. Did you have any practical experience of working with Patient Research Partners during EuroTEAM? If so, please describe your own positive and negative experiences: |
|  |
| 10. Can you suggest ways in which Patient Research Partner involvement in EuroTEAM could have been improved from your perspective? |
|  |
| 11. In what ways has the involvement of Patient Research Partners in EuroTEAM had an impact on how you would involve Patient Research Partners in future projects? |
|  |

**Thank you!**
